# Thermal conductance calculations of silicon nanowires: comparison with diamond nanowires

**DOI:** 10.1186/1556-276X-8-256

**Published:** 2013-05-29

**Authors:** Kohei Yamamoto, Hiroyuki Ishii, Nobuhiko Kobayashi, Kenji Hirose

**Affiliations:** 1 Institute of Applied Physics, University of Tsukuba, 1-1-1 Tennoudai, Tsukuba, Ibaraki 305-8573, Japan; 2 Green Innovation Research Laboratory, NEC Corporation, 34 Miyukigaoka, Tsukuba, Ibaraki 305-8501, Japan

**Keywords:** Thermal conductance, Nanowire, Silicon, Diamond, Non-equilibrium Green’s function

## Abstract

We present phonon thermal conductance calculations for silicon nanowires (SiNWs) with diameters ranging from 1 to 5 nm with and without vacancy defects by the non-equilibrium Green’s function technique using the interatomic Tersoff-Brenner potentials. For the comparison, we also present phonon thermal conductance calculations for diamond nanowires. For two types of vacancy defects in the SiNW, a ‘center defect’ and a ‘surface defect’, we found that a center-defect reduces thermal conductance much more than a surface defect. We also found that the thermal conductance changes its character from the usual behavior, in proportion to the square of diameter (the cross-sectional area) for over 100 and 300 K, to the unusual one, not dependent on its diameter at all at low temperature. The crossover is attributed to the quantization of thermal conductance.

## Background

Phonon thermal transport properties of silicon nanowires (SiNWs) have attracted much attention recently. As the size of silicon electron devices with nanowire structures becomes smaller and smaller to the nanometer scale, the thermal heating problem becomes serious. Also, SiNWs are regarded to be a good candidate for efficient thermoelectric devices.

Experimentally, thermal conductivities of SiNWs with diameters ranging from 22 to 115 nm [1] and from about 15 to 50 nm [2] have recently been measured and showed unusually low thermal transport properties. The measured thermal conductivities show different temperature dependence for different diameters of nanowires due to the confinement effects to nanometer size. To understand the thermal transport properties of SiNWs less than 100 nm in diameter, we need to consider the phonon problems from an atomistic point of view.

Theoretically, Mingo et al. [3] calculated thermal conductivities of SiNWs with diameters larger than 35 nm, using the phonon dispersion relation from the data of bulk silicon, and showed good agreement with the experiments. This shows that thermal conductance calculations with the Boltzmann transport formula or molecular dynamics calculations are effective at high temperature in diffusive regime. However, for phonon transport at low temperature or with diameters less than 30 nm, the effects of nanometer-scale structures such as confinement and low speed modes on phonon transport become significant [3]. For such regimes, we need the computational approach, taking the quantum effects explicitly into account. These effects for thermal transport can be included when we use the transmission approach, where the Landauer formula [4] or the non-equilibrium Green’s function (NEGF) technique based on the Keldysh’s theory has been widely studied [5]. The NEGF approach has been well established [6,7] for electron transport and also the formulation is derived for phonon transport [8]. Recently, some theoretical works have been performed based on the atomistic models using the NEGF technique to calculate the thermal conductances of SiNWs [9-11] and carbon nanotubes [12].

In the present work, we treat the thermal conductance of SiNWs in comparison to the diamond nanowires (DNWs) which have the same atomistic configurations but are made of the different atomic types. Since the bulk diamond has very high thermal conductivity, we expect that DNWs might also have high thermal conductivity. Here we use the NEGF technique with empirical Tersoff-Brenner interatomic potentials for the atomistic calculations of thermal conductance of SiNWs and DNWs. We present thermal conductance of SiNWs with diameters from 1 to 5 nm with and without vacancy defects and DNWs with diameters ranging from 1 to 4 nm without defects. The diameter dependences of thermal conductances of SiNWs and DNWs with no defects are presented for the temperature ranging from 0 to 300 K. We show how the thermal conductances of SiNWs and DNWs change their behaviors as the temperature decreases with their thickness.

First, for the ideally clean SiNWs and DNWs with no defects, we found that the thermal conductances of SiNWs and DNWs are proportional to their cross-sectional area for over 100 and 300 K, where the thermal conductance of pristine SiNW becomes much higher than that measured experimentally [2], which might have a number of defects. Thus, we present thermal conductance calculations of SiNWs with diameters from 1 to 2 nm with vacancy defects, focusing especially on the difference of the position of the vacancies, where we consider two types of a vacancy: a ‘surface defect’ with an atom at the surface is missing and a ‘center defect’ with an atom at the center of cross section of wires is missing for an example of a simple defect. We found that thermal conductance reduces much more for a center defect than for a surface defect. Finally, we compare thermal transport properties of SiNWs and DNWs and discuss the effects of differences of atomic types.

## Methods

**Figure 1:**
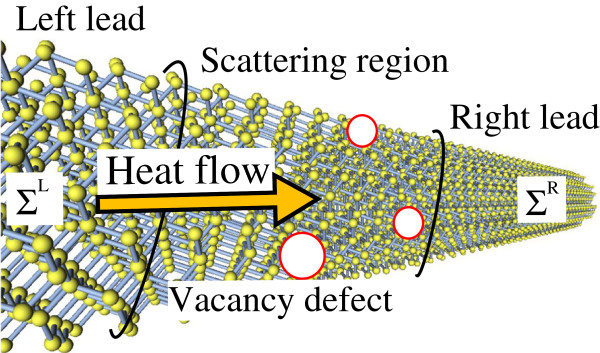
**Schematic view of the atomistic model of SiNW for *〈100〉 *direction with a diameter of 2 nm.** The system is divided into three parts by black lines: left lead, scattering region, and right lead. Vacancy defects are introduced in the scattering region, while no defects are present in the left and right leads. Red circles represent the vacancy defects.

We split the total Hamiltonian into four pieces: *H*=*H*_L_+*H*_S_+*H*_R_+*H*_int_, where *H*_L(R)_ is the Hamiltonian for the left (right) lead, *H*_S_ is for the scattering region, and *H*_int_ is for the interaction between the scattering region and the left(right) lead (Figure 1).

The thermal current *J*_th_ from the left lead to the scattering region can be expressed by the following formula with the NEGF technique [12]

(1) $$J_{\text{th}}=-\langle \dot{H_{L}}\rangle =\int_{0}^{\infty }\mathrm{d\omega \hbar \omega}\left[n(\omega ,T_{L})-n(\omega ,T_{R})\right]\;\zeta \;(\omega ).$$

Here the bracket 〈...〉 denotes the non-equilibrium statistical average of the physical observable, *n*(*ω*,*T*_L(R)_) is the Bose-Einstein distribution function of equilibrium phonons with an energy of ℏω in the left (right) lead at temperature *T*_L(R)_. *ζ*(*ω*) is the transmission coefficient for the phonon transport through the scattering region given by

(2) $$\zeta (\omega )=\text{Tr}\;\left[\Gamma_{L}(\omega )G^{r}(\omega )\Gamma_{R}(\omega )G^{a}(\omega )\right].$$

Here, *G*^r/a^(*ω*) is the retarded (advanced) Green’s function for the scattering region and *Γ*_L/R_(*ω*) is the coupling constant.

In the limit of small temperature difference between left and right regions, the thermal conductance *G* is given by

(3) $$\;G(T)\;=\;\frac{dJ_{\text{th}}}{\text{dT}}\;=\;\frac{\hbar^{2}}{2\pi k_{B}T^{2}}\;\;\int_{0}^{\infty }\mathrm{d\omega \zeta}(\omega )\omega^{2}\frac{e^{\hbar \omega /k_{B}T}}{{\left(e^{\hbar \omega /k_{B}T}-1\right)}^{2}}.$$

For the ideal ballistic limit without any scattering, *ζ*(*ω*) is equal to the number of phonon subbands at frequency *ω*.

The retarded (advanced) Green’s function for the scattering region is given by

(4) $$G^{r/a}(\omega )={\left[\omega^{2}M-K-\Sigma_{L}^{r/a}-\Sigma_{R}^{r/a}\right]}^{-1},$$

where *M* is the diagonal matrix whose element is a mass of atom and $\Sigma_{L/R}^{r/a}(\omega )$ is the retarded (advanced) self-energy due to the coupling to the left (right) semi-infinite lead with the scattering region, which is obtained independently from the atomistic structure of the lead. We use a quick iterative scheme with the surface Green’s function technique [13] to calculate the self-energy for complex atomic structures of SiNWs. The coupling constant *Γ*_L/R_(*ω*) in Equation 2 is then obtained from

(5) $$\Gamma_{L/R}(\omega )=i[\Sigma_{L/R}^{r}(\omega )-\Sigma_{L/R}^{a}(\omega )].$$

The total Hamiltonian for the phonon is determined from the dynamical matrix *K*, which is constructed from the force constants between the atoms. The matrix elements of *K*_*iα*,*jβ*_ are calculated by finite difference of the force *F*_*iα*_ with respect to *r*_*jβ*_ such as

(6) $$K_{\mathrm{i\alpha},\mathrm{j\beta}}=\frac{\partial^{2}E}{\partial r_{\mathrm{i\alpha}}\partial r_{\mathrm{j\beta}}}=\frac{-[F_{\mathrm{i\alpha}}(+\Delta R_{\mathrm{j\beta}})-F_{\mathrm{i\alpha}}(-\Delta R_{\mathrm{j\beta}})]}{2\Delta R_{\mathrm{j\beta}}}.$$

The force *F*_*iα*_ is obtained from the derivative of *E* with respect to *r*_*iα*_ where *E* is the total energy of the system and *r*_*iα*_ is the atomic coordinate of the *i*th atom along the *α* direction. Therefore *F*_*iα*_(+*ΔR*_*jβ*_) indicates the force of *i*th atom along the *α* direction generated by the *j*th atom along the *β* direction with a displacement of +*ΔR* from the pristine wire’s equilibrium positions. Here *ΔR* is a displacement, for which we take *ΔR*=2×10^−4^Å in the present work.

As for the total energy formula *E*, we use the interatomic Tersoff-Brenner potential [14,15] for silicon and carbon atoms. Here we note that according to the recent calculation for the thermal conductance of SiNWs with no defects and with edge atoms passivated by hydrogen, the force constants calculated by the *ab initio* density functional theory for H-passivated SiNW produce almost the same thermal conductance with those obtained from the interatomic Tersoff potential without H passivation [11]. Therefore, we employ here the interatomic Tersoff potential for SiNW.

## Results and discussion

**Figure 2:**
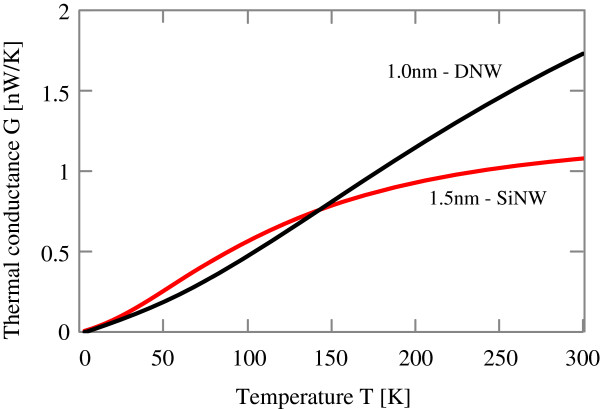
**Thermal conductance of SiNW and DNW.** Red and black solid lines show thermal conductances of 〈100〉 SiNW with 1.5 nm in diameter and 〈100〉 DNW with 1.0 nm in diameter.

First, let us see the temperature dependence of thermal conductance. Figure 2 shows the thermal conductance of a SiNW with 1.5 nm in diameter and that of a DNW with 1.0 nm in diameter as a function of temperature. Here, no defects are present for these two wires to see the temperature dependence of thermal conductance clearly. Generally, thermal conductance is zero at 0 *K* because no phonons are excited for the propagation of heat. With temperature increases, the thermal conductance increases monotonically without any scatterings and saturates at high temperature, where the dependence changes from material to material. This monotonic increase of thermal conductance reflects the phonon occupation according to the Bose-Einstein distribution and is quite different from the electron conductance in which only a small number of electrons around Fermi level contributes to the conduction. We note that the behavior at high temperature near the saturation is determined by the highest phonon energy of each material, which is observed in the phonon band structure. For SiNW case, the thermal conductance starts to saturate around 300 K, because almost all phonons of SiNW are excited for thermal conduction at around 300 K. We can see that the DNW with 1.0-nm diameter has a higher thermal conductance than the SiNW with 1.5 nm at the temperature higher than 150 K. For the DNW, the thermal conductance starts to saturate around 800 K, which is also determined by the highest phonon energy as can be seen in the phonon band structure of the DNWs.

**Figure 3:**
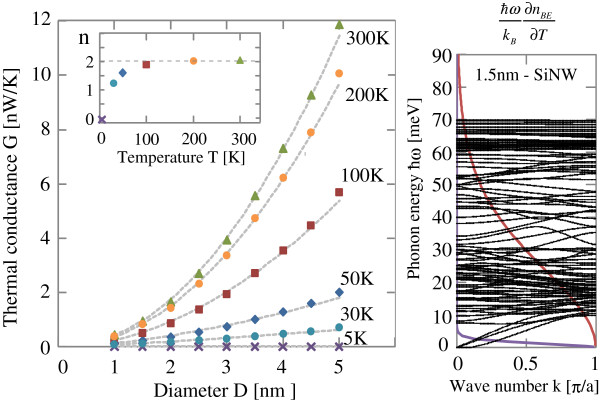
**Thermal conductance of SiNW and phonon dispersion relation.** Thermal conductance as a function of the diameter of SiNW without vacancy defects for several temperature. Inset is the exponent n of diameter dependence of thermal conductance for several temperature. (right) Phonon dispersion relation of 〈100〉 SiNW with 1.5 nm in diameter for the wave vector *q*. Here *a*=5.362 Å. Red and purple solid lines show weight functions in thermal conductance for 100 and 5 K.

The left panel of Figure 3 shows the thermal conductance of SiNWs as a function of the diameter at various temperatures from 5 K up to 300 K, and the inset shows an exponent *n* of the diameter dependence of thermal conductance as defined by *G*∝*L*^*n*^. We see that the quantized thermal conductance, which does not depend on the wire diameter, appears below 5 K. With increasing temperature, the thermal conductance comes to depend on its diameter. For over 100 K, we see that the thick SiNW with a large diameter has a larger thermal conductance proportional to the cross-sectional area, which reflects its atomic structure since the SiNW has the columnar shape and the total number of silicon atoms in the SiNW is proportional to its cross-sectional area. This indicates that the thermal conductance in the defect-free clean limit is determined by the total number of atoms in the nanowire structures. The right panel of Figure 3 shows the phonon dispersion relation of 〈100〉 SiNW with 1.5 nm in diameter. We see that the phonon dispersion of SiNW spreads up to 70 meV, which is determined by the interaction between silicon atoms. As the thickness of the wire becomes larger and larger, the number of phonon subbands increases in proportion to the number of silicon atoms.

**Figure 4:**
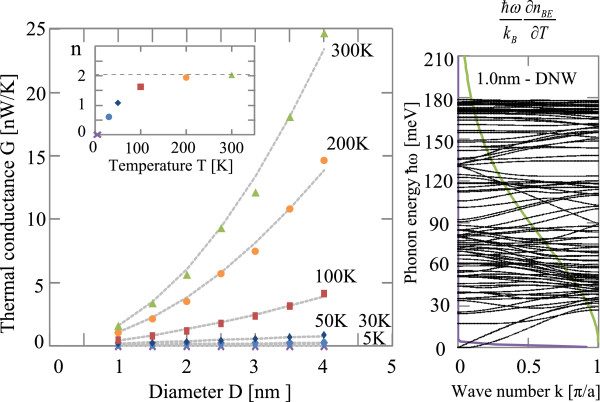
**Thermal conductance of DNW and phonon dispersion relation.** (left) Thermal conductance as a function of the diameter of DNW without vacancy defects for several temperature. Inset is the exponent *n* of diameter dependence of thermal conductance for several temperature. (right) Phonon dispersion relation of 〈100〉 DNW with 1.0 nm in diameter for the wave vector *q*. Here *a*=3.567 Å. Green and purple solid lines show weight functions in thermal conductance for 300 and 5 K.

The left panel of Figure 4 shows the thermal conductance of DNWs as a function of the diameter at various temperatures from 5 K up to 300 K, and the inset shows an exponent of the diameter dependence of thermal conductance. Similarly as in Figure 3, we can see the quantized thermal conductance below 5 K and the thermal conductance comes to depend on its diameter with an increase of temperature. We also see that the thick wire with the large diameter has the larger thermal conductance, which is proportional to the cross-sectional area of the DNW at the temperature over 300 K. Since the DNW also has the columnar shape, the total number of carbon atoms in the DNW is also proportional to its cross-sectional area. Then, we can say that the thermal conductance of DNW in the defect free-clean limit is determined by the total number of atoms in the nanowire structures. The right panel of Figure 4 shows the phonon dispersion relation of 〈100〉 DNW with 1.0 nm in diameter. We see that the phonon dispersion of DNW spreads up to 180 meV, which is determined by the interaction between the carbon atoms. As the thickness of the wire becomes larger and larger, the number of phonon subbands also increases in proportion to the number of carbon atoms.

Next, let us consider the effects of difference of atomic types. Since atomistic configurations are the same for SiNW and DNW, the phonon band structures of SiNW and DNW are similar. The difference of phonon bands is only the highest phonon energy. Namely, the phonon band of SiNW spreads from 0 meV up to 70 meV, while the phonon band of DNW spreads from 0 meV up to 180 meV. This leads to the difference of saturation temperature of thermal conductance. With an increase of temperature, phonons which have higher energies are excited and propagate heat gradually, thus the thermal conductance increases gradually. As a result, the thermal conductance increase of DNW remains for higher temperature compared with that of SiNW. That is why the DNW with 1.0 nm width has a higher thermal conductance than the SiNW with 1.5 nm width for over 150 K. For the temperature less than 150 K, the SiNW with 1.5 nm width has a larger number of phonons which propagate heat more than the DNW and thus the SiNW has a higher thermal conductance. Moreover, the difference of the highest phonon energy leads to the difference of crossover temperature. As shown in the insets of left panels of Figures 3 and 4, the exponents *n* are 0 at 0 K and with an increase of temperature, *n* of SiNW approaches *n*=2 at around 100 K while that of DNW becomes *n*=2 at around 300 K. Here we note that when the exponent becomes *n*=2, the thermal conductance of wire is proportional to its cross-sectional area, since the number of atoms of the wire is proportional to its cross-sectional area. For the SiNW, at around 100 K, all the phonons of SiNW propagate heat and the thermal conductance becomes proportional to the total number of phonons. Since the total number of phonons is equal to the product of 3 times the number of atoms, the thermal conductance is proportional to the number of atoms of wire at around 100 K. On the other hand, for the DNW, all the phonons propagate heat at around 300 K and the exponent *n* becomes *n*=2 at around 300 K.

**Figure 5:**
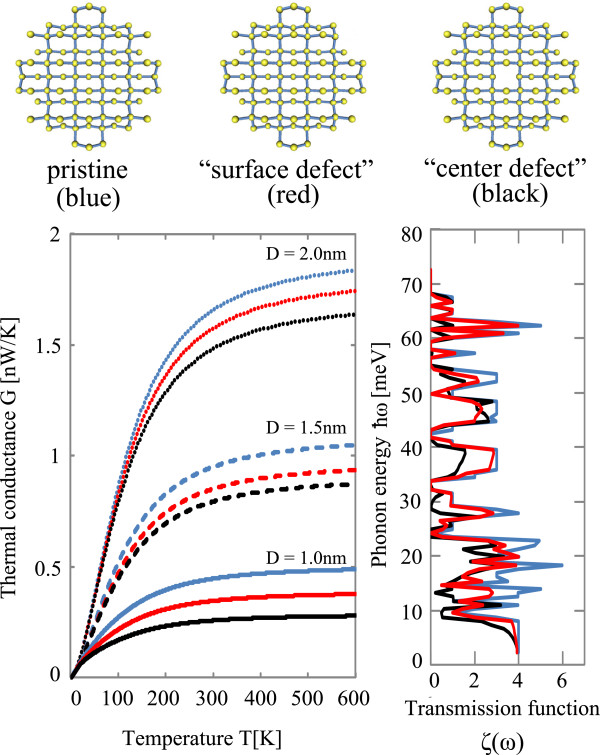
**Thermal conductance and transmission coefficients of SiNW with defects.** (top) Atomistic models of 〈100〉 SiNW with 2 nm in diameter with no defects (top-left), a surface defect (top-middle), and a center defect (top-right). The wire is oriented along the perpendicular direction to the sheet. (bottom-left) Temperature dependence of thermal conductance of SiNWs with no defects (black lines), a surface defect (blue lines), and a center defect (red lines), for various diameters of *D*=1.0 nm (solid lines), *D*=1.5 nm (dashed lines), and *D*=2.0 nm (dotted lines), respectively. (bottom-right) Transmission coefficients of the SiNWs with no defects (black lines), a surface defect (blue lines), and a center defect (red lines), respectively, for 1.0 nm in diameter.

The lower left panel of Figure 5 (black lines) shows the thermal conductance of SiNW as a function of temperature. It should be noted that recent experiments for SiNWs with larger diameter than about 30 nm [1,2] show that the thermal conductance drops down in the high-temperature region, which might be caused by the anharmonic effects, missing in the present work, as suggested by Mingo et al. [3] from the classical conductance calculation. When we compare the above calculations with the experiment [2], which has measured thermal conductance of about 2 nW/K for SiNW with 15 nm in diameter at 300 K, we found that the calculated thermal conductance of SiNW takes more than 2 nW/K even for 3 nm in diameter at 300 K. This clearly shows that the assumption of no defects overestimates the thermal conductance of SiNW and thus understanding of the effects of defects is essential for the thermal transport of SiNW. Actually, the phonon-phonon scatterings due to anharmonic effects are not important for SiNWs with diameters smaller than 30 nm [3]. Then, for one of the simplest defects, we introduce a single vacancy. Markussen et al. have studied the effect of surface vacancy defects by taking sample average of SiNWs with randomly placed surface vacancies [16,17]. Here we focus on the effect of a vacancy at different positions on the thermal conductance.

The bottom-left panel of Figure 5 shows the temperature dependence of the thermal conductance with no defects, with a surface defect, and with a center defect for three diameters *D* = 1.0, 1.5, and 2.0 nm. Since the phonon-phonon scatterings due to anharmonic effects are not taken into account here, the thermal conductance drop observed in the high temperature regime in experiments [1] for a thick SiNW with a diameter larger than 30 nm is not reproduced and is different from the previous work [3]. As for the effects of vacancy defects on the thermal conductance, we can see that for all diameters of SiNWs and all temperature regions, the pristine wire has the highest thermal conductance, and the vacancy effects are more significant for a center defect than for a surface defect. It would be interesting to investigate why the SiNWs have different thermal conductances when defects are included at different positions. It looks like the effects of vacancy defects on the thermal conductance are not simple, since we cannot estimate the behaviors only from the density of vacancy defects. To understand the effects of vacancy defects, we have to take the calculated results of atomistic transmission functions into account. The bottom-right panel of Figure 5 shows the transmission coefficients *ζ*(*ω*) for the SiNWs with 1.0 nm in diameter with no defect (black line) and a vacancy defect (blue and red lines). We can see that the transmission coefficient decreases much more for the SiNW with a center defect than that with a surface defect at several specific energies. This result is related to the details of phonon modes with specific energies. In those modes, the center atom has an important role in the vibration modes while the corresponding edge atom is not so important. This effect on the phonon mode causes different behaviors of thermal conductance between a center defect and a surface defect for thin SiNWs.

## Conclusions

To conclude, we have applied the NEGF technique with the interatomic Tersoff-Brenner potential for the phonon thermal transport of SiNWs with and without a vacancy defect and DNWs with no defects. We found that crossover from the quantized thermal conductance to the usual thermal conductance appears with increasing temperature from 5 K up to 300 K for both SiNW and DNW. We also found that thermal conductances of SiNW and DNW with no defects were in proportion to their cross-sectional area for 100 and 300 K. This reflects the columnar shape of SiNW and DNW. Compared with the recent experiments, understanding of the effects of defects is essential for thermal conductance of SiNWs. We found that a center defect reduces the thermal conductance much more than a surface defect. This is due to the effects on the specific phonon modes where a center atom has various covalent bonds with neighbor atoms while an edge atom does not have. This concludes that the effects of vacancy defects on the thermal conductance of nanometer-size SiNW are not simply estimated from the density of vacancy defects, but instead we have to take the effects of vacancy defects on the thermal conductance from precise atomistic structures into account.

## Competing interests

The authors declare that they have no competing interests.

## Authors’ contributions

KY carried out the calculation, and drafted the manuscript. HI participated in the discussion. NK supervised the study and KH advised on the work. All authors read and approved the final manuscript.
